# Current advances in serological and molecular diagnosis of *Schistosoma mekongi* infection

**DOI:** 10.1186/s41182-024-00598-0

**Published:** 2024-04-22

**Authors:** Adrian Miki C. Macalanda, Atcharaphan Wanlop, Kevin Austin L. Ona, Eloiza May S. Galon, Virak Khieu, Somphou Sayasone, Aya Yajima, Jose Ma. M. Angeles, Shin-ichiro Kawazu

**Affiliations:** 1 Department of Immunopathology and Microbiology, College of Veterinary Medicine and Biomedical Sciences, Cavite State University, Indang, 4122 Cavite, Philippines; 2 National Research Center for Protozoan Diseases, Obihiro University of Agriculture and Veterinary Medicine, Obihiro, Hokkaido 080-8555 Japan; 3 Department of Parasitology, Institute of Tropical Medicine, Nagasaki University, Nagasaki, 852-8131 Japan; 4 College of Medicine, The University of the Philippines – Manila, Pedro Gil St., Ermita Manila, Manila, Philippines; 5 National Center for Parasitology, Entomology and Malaria Control, Phnom Penh, Cambodia; 6 Lao Tropical and Public Health Institute, Vientiane, Lao PDR; 7 World Health Organization Regional Office for Southeast Asia, New Delhi, India; 8 Department of Parasitology, College of Public Health, The University of the Philippines - Manila, Pedro Gil St., Ermita Manila, Manila, Philippines

## Abstract

Schistosomiasis, a neglected tropical disease, caused by blood flukes belonging to the genus *Schistosoma;* it persists as a public health problem in selected regions throughout Africa, South America, and Asia. *Schistosoma mekongi,* a zoonotic schistosome species endemic to the Mekong River in Laos and Cambodia, is one of the significant causes of human schistosomiasis along with *S. japonicum, S. mansoni, S. haematobium* and *S. intercalatum*. Since its discovery, *S. mekongi* infection has been highly prevalent in communities along the Mekong River. Although surveillance and control measures have shown success in recent years, more robust diagnostic tools are still needed to establish more efficient control and prevention strategies to achieve and sustain an elimination status. Diagnosis of *S. mekongi* infection still relies on copro-parasitological techniques, commonly made by Kato-Katz stool examination. Serological techniques such as enzyme-linked immunosorbent assay (ELISA) may also be applicable but in a limited setting. Targeted molecular and serological tools specific to the species, on the other hand, have been limited. This is due, in part, to the limited research and studies on the molecular biology of *S. mekongi* since genome information of this species has not yet been released. In this review, current advances, and gaps and limitations in the molecular and immunological diagnosis of *S. mekongi* are discussed.

## Introduction

Human schistosomiasis is a neglected tropical disease caused by an infection with the helminthic parasite *Schistosoma* spp. Affecting approximately 250 million people worldwide, schistosomiasis has persisted as a public health concern in selected regions throughout Africa, South America, and Asia [17]. There are three known species of schistosomes affecting the Asian region: *S. japonicum, S. mekongi*, and *S. malayensis* [17, 40, 59]. Among the three, *S. mekongi* will be the focus of this review.

*S. mekongi* is a zoonotic schistosome species endemic to the Mekong Delta, particularly in the northern region of Cambodia and the southern region of the Lao People’s Democratic Republic [17, 41]. Its endemic distribution is limited to the areas inhabited by *Neotricula aperta* the intermediate host snail, whose range is limited by specific ecological conditions [3, 27, 47, 53]. Similar to its closely related species *S. japonicum*, *S. mekongi* may likewise infect a variety of domestic animals including pigs, water buffalo, and cattle [59].

The main control measures done in schistosomiasis-endemic areas mainly rely on the use of the anthelmintic drug praziquantel (PZQ) in a mass drug administration (MDA) scheme [15]. Through improved sanitation throughout Southeast Asia as well as sustained efforts using MDA, the prevalence of schistosomiasis mekongi has decreased considerably through the years [17]. The continuous decline in the intensity of infection has further resulted in a decrease in the sensitivity of stool microscopy [13]. That, together with the variation in egg deposition of adult worms, considerably hinders the ability to detect infection [72].

Accurate diagnosis is an indispensable tool for monitoring treatment success as well as case detection to determine disease prevalence. To address the current difficulty faced using traditional stool microscopy, several studies have been done to detect *S. mekongi* infection. Modern molecular biological techniques based on parasite-derived biomarkers and nucleic acid as well as host-derived antibodies indicative of infection has shown promising results [9, 25, 31, 49, 59, 65]. Here, we review the current molecular and serological methods in detecting *S. mekongi* infection and its potential contribution towards the successful elimination of schistosomiasis mekongi.

## Parasite biology

The life cycle of *S. mekongi* starts when an infected human or animal defecates near a body of water. The egg further develops into an embryonated egg and, upon hatching in the water, the miracidium swims out and seeks the first intermediate host, *N. aperta, *an aquatic snail that belongs to the Pomatiopsidae family. Inside the snail, the miracidium develops further into a sporocyst, where, through clonal replication, numerous cercariae are released into the water. The cercaria through the action of proteases can penetrate the intact skin of the host gaining access to the vasculature. The cercaria loses its tail upon penetration and develops into a schistosomulum. The schistosomulum swims into the blood vessel until it reaches the veins of the mesentery and the portal vein of the liver. The fluke further matures into an adult, where the female copulates with a male schistosome in the blood vessel resulting in the fertilization and eventual deposition of eggs. The eggs cause granulomatous lesions in the endothelium of blood vessels as well as the organs in which the eggs get trapped. The eggs that end up in the intestinal lining cause ulceration to the epithelium which leads to the liberation of the egg into the lumen of the intestine hence releasing the eggs in the feces [42].

## History, distribution and hosts

The discovery of *S. mekongi* dates back to the mid-twentieth century when a team of scientists from the World Health Organization (WHO) and the Rockefeller Foundation began investigating cases of schistosomiasis in Cambodia and Laos (Word Health Organization, 2020). In 1957, a new species of *Schistosoma* was discovered in the blood vessels of infected individuals living near the Mekong River [14, 62]. *S. mekongi* is one of the five species of *Schistosoma* that are known to infect humans, and it is primarily found in Southeast Asia, specifically in the Mekong River basin [27]. The new species was later named *S. mekongi* after the river where it was first discovered. Since its discovery, *S. mekongi* has been recognized to be a major public health concern in Southeast Asia, where it has been responsible for causing outbreaks of schistosomiasis [47]. The disease is transmitted through contact with contaminated freshwater, which contains the cercariae of the parasite [63]. Once inside the body of humans or animals, the larvae mature into adult worms in the mesenteric veins causing a range of symptoms, including abdominal pain, diarrhea, and liver damage [8]. Efforts to control and eliminate schistosomiasis caused by S*. mekongi* have been ongoing for several decades. These efforts have included the use of PZQ, a drug that is effective at killing adult worms and reducing the severity of symptoms. Other strategies have included improving sanitation and hygiene, providing access to safe drinking water, and controlling the population of *N. aperta* which serves as the intermediate host for this parasite. Despite these efforts, schistosomiasis remains a significant public health concern in Southeast Asia, with thousands of people still being infected each year. However, sustained efforts and collaboration among public health organizations and governments in the region offer hope for the eventual elimination of *S. mekongi* and other species of *Schistosoma* that cause schistosomiasis [47, 63].

## Epidemiology

*S. mekongi* has been targeted for control and elimination in Cambodia and the Lao People's Democratic Republic (PDR) through a combination of MDA using PZQ, health education, and improvements in water and sanitation infrastructure. In 2015, the prevalence of *S. mekongi* infection in the Lao PDR has declined significantly and in recent years following these efforts. Furthermore, it was found that the prevalence of the infection among school-aged children decreased from 31.3% in 2007 to 9.5% in 2012, while the overall prevalence of the infection in the general population decreased from 7.5% in 2007 to 2.5% in 2012. Similarly, in Cambodia, the prevalence of *S. mekongi* infection among school-aged children decreased from 3.3% in 2006 to 0.3% in 2012 following the implementation of control measures. These studies suggest that control efforts have been effective in reducing the prevalence of *S. mekongi* infection in affected areas. As such, sustained efforts at detecting infection and treatment are needed to eventually eliminate schistosomiasis caused by *S. mekongi* [27, 41].

## Diagnosis of *S. mekongi* infection

### Parasitological detection

Diagnostic techniques with high sensitivity and specificity are key to the control and elimination of schistosomiasis. Currently, a simple and cheap method with high level of sensitivity and specificity for *S. mekongi* diagnosis is not yet available. Parasitological detection are the most commonly used techniques in endemic areas, and these involve examining stool samples for parasite eggs using various methods such as direct fecal smear, Kato-Katz technique, miracidium hatching test, formalin–ether concentration technique and fecal sedimentation [12, 18, 26, 47, 63, 66]. These techniques are cost-effective and relatively easy to perform [6]. During a schistosome’s life cycle, the eggs are released by the adult worms within the definitive host. This indicates that parasitological methods can detect active infection by detecting the presence of eggs [40]. Furthermore, these approaches are highly specific in areas with high prevalence and intensity of infection. However, its sensitivity becomes a problem in endemic areas with light infections or after PZQ treatment which may eventually lead to a misdiagnosis [33, 54, 56, 58]. Although repeated screening of individual samples may increase sensitivity, it would be costly and time-consuming [1]. Therefore, more accurate methods such as molecular and immunological detection are needed for monitoring the true prevalence of *S. mekongi* infection in the endemic areas.

### Molecular-based diagnosis

Nucleic acid detection techniques have become more popular over the years. These methods are commonly DNA-based and take advantage of the DNA’s presence in a sample collected from the host. In general, DNA-based methods, particularly the polymerase chain reaction (PCR) technique, has high sensitivity and accuracy and can be designed to be specific to a genus or species [36, 37]. These methods can distinguish species of *Schistosoma* from one another [32, 50, 64]. Nucleic acid such as DNA of schistosomes can be released to the host’s blood circulation upon entry and during the parasite’s development [7, 71]. Hence, the nucleic acid detection is useful in diagnosing active schistosome infection [25, 59]. [55] likewise demonstrated that schistosome DNA can be detected in experimentally infected mice before parasite eggs are demonstrated on the feces. In addition, stool PCR targeting the 18S rRNA gene of *S. mekongi* was successfully used and was able to detect a single egg in a stool sample [29]. These methods, however, might not be as effective when used post-treatment [59, 64]. In general, PCR and PCR-coupled techniques are known to be time-consuming and laborious, and require expensive equipment making them not suitable for point-of-care diagnosis and broad surveillance studies [32, 37].

To date, the use of nucleic acid detection techniques in *S. mekongi* diagnosis has been very limited primarily due to non-availability of its whole genome sequence. Conventional PCR is the one more popularly used in diagnosing parasitic infections. Using primers targeting a specific gene, a parasite species can be identified and differentiated from another. Conventional PCR, however, requires analysis by agarose gel electrophoresis and subsequent visualization which are both contaminant-prone, time-consuming, and laborious [32, 37, 52].

Not many studies that used conventional PCR for detecting *S. mekongi* infection have been reported in the past few years [49]. Kato-Hayashi et al. evaluated the use of mitochondrial gene *cytochrome c oxidase subunit 1* (*cox1*) in conventional PCR for major schistosome species including *S. mekongi*. The authors showed that each species-specific primer pair was able to detect presence of schistosome DNA even in the case with DNA content as little as 0.1 pg, and does not cross amplify the DNA of other species when used in multiplex PCR. In addition, the PCR protocol was able to detect the presence of schistosome DNA as early as one day post-infection.

#### RT-PCR and PCR-coupled techniques

Real-time (RT)-PCR and other PCR-coupled techniques make analysis of results faster and more efficient and generally do not require post-PCR processing like gel electrophoresis. Cnops et al. developed a genus-specific RT-PCR to detect major schistosome species including *S. mekongi* in infected travelers using fecal and urine samples. Targeting the 28S ribosomal RNA gene, the protocol showed high sensitivity and specificity, and did not show cross reactivity [9]. Using species-specific primers, similar protocols were developed for detection of *S. mekongi* infection*.*

Wittwer et al. developed high-resolution melting analysis (HRM) RT PCR which showed potential in parasite detection and its subsequent identification [67]. This PCR-coupled technique uses a closed-tube system that permits direct and rapid analysis of probe-free RT PCR products wherein their specific melting curves are accurately determined [32, 43]. This technique is simpler, more efficient and less costly, and allows rapid and sensitive screening of the infection, making it suitable for large-scale applications [29, 32]. HRM real-time PCR targeting the V4 region of 18S ribosomal RNA gene was able to distinguish *S. mekongi* from the others. In addition, *S. mekongi* showed a similar melting curve peak with *S. japonicum* in this assay at 83.65 °C; however, they could be discriminated by their profiles [32]. The results were consistent with the study by Konglieng et al*.* using the same method to differentiate *S. japonicum* from *S. mekongi* in which sensitivity and specificity in the differential detection of them were both 100%.

#### LAMP

Loop-mediated isothermal DNA amplification (LAMP) is a simple and non-PCR nucleic acid detection method. It offers high sensitivity and specificity together with an efficient and fast quantitative detection of species-specific target genes [37, 45]. It is suitable for diagnosing low intensity infection [34]. This technique does not require a thermal cycler, and uses a thermal block or water bath instead, thus making it suitable for field application [45].

LAMP has been used for field surveillance and monitoring, and patient diagnosis of *Schistosoma* spp. [16, 19, 30, 31, 34]. LAMP protocol targeting the Internal transcribed spacer 1 (ITS1) gene was used for detecting *S. mekongi* infection using stool samples from individuals living in the disease-endemic village in Laos. The protocol showed sufficiently high sensitivity and specificity, and could detect the target DNA in amount as low as 1 pg. The protocol could detect 2.9% of the samples as positive, while Kato-Katz could detect 0.4% (Tables 1 and 2) of the samples [31].

**Table 1:** Summary of molecular-based methods used for the detection of *S. mekongi* infection

Diagnostic platform	Gene target	Type and source of sample used for testing	References
Conventional PCR	Cytochrome C oxidase 1	Mouse or Mongolian gerbils’ serum	[25]
Real-time PCR	Mitochondrial genome	Rat feces	[52]
	28 s rRNA	Human feces	[9]
	18 s rRNA	Whole parasite—from experimentally infected rats	[29]
	V4 region 18 s rDNA	Whole parasite—from experimentally infected rabbit or mice	[32]
PCR-coupled with pyrosequencing	18 s rRNA	Rat feces	[57]
LAMP	Internal transcribed spacer 1	Human feces	[31]

**Table 2:** Summary of serological based methods used for the detection of *S. mekongi* infection

Diagnostic platform	Source and name of antigen target	Type and source of sample used for testing	Reference(s)
Antibody ELISA	*Megathura crenulata* keyhole limpet haemocyanin, *S. mekongi* adult worm antigen and *Trichinella spiralis* excretory-secretory antigen	Human serum	[23]
	*S. mekongi* soluble egg antigen	Human serum	[28]
	*S. mansoni* adult worm antigen extract	Human serum	[44, 63]
	*S. mekongi* thioredoxin peroxidase-1	Human serum	[65]
	*S. japonicum* crude soluble egg antigen, Tandem repeat protein, Phytochelatin synthase, Peroxiredoxin-4 and Saposin + TPx-1 + LHD chimera	Human serum	[2]
	*S. japonicum* soluble egg antigen	Human serum	[73]
Antigen ELISA	*S. mansoni* circulating cathodic antigen (CCA)	Human urine	[61, 63]
	*S. mansoni* circulating anodic antigen (CAA)	Human urine	[61, 63]
	*S. mekongi* kyphoscoliosis peptidase and putative tuberin	Mice serum	[59]

## Serological diagnosis

### Antibody detection

Antibody-based serological assay has been proven to be beneficial in cases of low endemic areas addressing the pitfalls of stool microscopy. Although considered as the gold standard for schistosomiasis diagnosis, microscopy is laborious and suffers low sensitivity in areas where prevalence of the disease is low. Antibody-based serological assay for *S. mekongi* may, therefore, contribute to the surveillance efforts directed towards case identification and eventual disease elimination [2, 38].

The first study to assess the serodiagnostic capability of different antigens to detect schistosomiasis mekongi used several antigens derived from several organisms. The following are the: keyhole limpet hemocyanin from *Megathura crenula*, *S. mekongi* adult worm antigen and an antigen derived from *Trichinella spiralis* co-immunoprecipitated by monoclonal antibody. These antigens demonstrated varying sensitivity and specificity. Among these, however, the *S. mekongi* adult worm antigen is the most promising with 100% sensitivity with a low specificity of 35% [23]. Similarly, other studies utilized antigens derived from a different but closely related species *S. japonicum*. The studies evaluated the performance of soluble egg antigen (SjSEA), while the other compared the SjSEA with several recombinant antigens derived from *S. japonicum.* Zhu et al. used two ELISA platforms, dipstick dye immunoassay (DDIA) and the conventional ELISA. High sensitivity and specificity were observed in cases of moderate infection using both DDIA and ELISA giving the same sensitivity and specificity of 99% and 100%. In case of low infection, both the DDIA and ELISA have a slightly lower sensitivity and specificity at 97% and 100%, respectively. Angeles et al. reported a comparable sensitivity of 96.4% and a specificity of 93.5% using 2 µg of SjSEA. While using the recombinant antigens from *S. japonicum* have varying degree of sensitivity and specificity. In addition, a crude adult worm antigen derived from *S. mansoni* showed a sensitivity of 95% [44]. Due to the lack of *S. mekongi*’s whole genome sequence, cloning and expression of native *S. mekongi* antigens is challenging. As such, the excellent sensitivity and specificity shown by antigens derived from other schistosome species may still prove useful for the serodiagnosis of schistosomiasis mekongi.

In contrast, Kirinoki et al. showed exceptional sensitivity and specificity using SEA derived from *S. mekongi* itself. Two ELISA platforms were used: conventional and sodium metaperiodate (SMP) ELISA. The sensitivity and specificity derived from conventional ELISA is 98% and 98%, respectively. While SMP–ELISA yielded a higher sensitivity of 100% and a specificity of 98%. Furthermore, the use of a native recombinant *S. mekongi* thioredoxin peroxidase-1 antigen showed promising results with a sensitivity and specificity of 89.3% and 93.3%, respectively. Serological tests detecting the antibody of host against *S. mekongi* may have several advantages over other diagnostic methods. It has acceptable sensitivity as well as potential applicability in the diagnosis of early stages of infection where traditional stool microscopy may be less sensitive as it was shown in schistosomiasis japonicum. Due to the continuous decline in the prevalence and the worm burden of patients infected with *S. mekongi*, antibody detection test may become valuable in such setting with near-elimination level of the disease [2, 39, 65].

## Antigen detection

In contrast to antibody detection-based assays, an antigen detection-based test may prove useful as it can detect current infection as well as evaluation of the response to cure [38]. However, such test has not yet been developed for the diagnosis of *S. mekongi* infection. A recent study by Uthailak et al. demonstrated potential circulating antigens that may be used for an antigen-based serodiagnostic assay detecting schistosomiasis mekongi. These antigens include kyphoscoliosis peptidase and putative tuberin. These antigens were detected at several time points in the infection; most notably during the early stages at 12 days post infection of experimentally infected mice. Due to the lack of *S. mekongi*-derived antigen-based test, circulating cathodic antigen (CCA) and circulating anodic antigen (CAA) derived from *S. mansoni* had been evaluated for detecting *S. mekongi* infection as a proof-of-concept study [61].

## CCA

The CCA has been well-studied as a proven antigen secreted by adult *S. mansoni* [11]. As there has been no antigen-based test detecting authentic *S. mekongi* CCA, a proof-of-concept study done in 2015 by van Dam et al. with the test detecting CCA derived from *S. mansoni* was able to detect 27 out of 69 Kato-Katz-positive individuals having a sensitivity of 39.0%. However, a more recent study done in Champassak utilizing the *S. mansoni* POCT–CCA kit could detect 11.5% of individuals positive out of 366 individuals sampled. Further, only one of the two Kato-Katz positive patients was POCT–CCA positive [21]. This result was much lower than that of the previous study by van Dam et al. which may indicate that the prevalence of *S. mekongi* is continually decreasing. The test with authentic CCA antigen derived from *S. mekongi* should be developed to overcome such limitations in the sensitivity and specificity of the existing test.

## CAA

Similarly, CAA of *S. mansoni* is a positively charged glycosaminoglycan regurgitated by the adult worm [10]. Among limited studies regarding CAA detection for *S*. *mekongi* infection, the study utilizing urine samples for detection of CAA derived from *S. mansoni* was able to detect 27 out of 69 Kato-Katz-positive individuals which showed comparable sensitivity to POCT–CCA of 39.0% [61]. In a similar study, a test kit used to detect CAA derived from *S. mansoni* from urine sample was able to detect 38.7% of the study participants. However, when serum samples were used, the prevalence rate decreased to 32.4%. The combined application of both urine and serum samples for diagnosis showed higher prevalence rate of 43.2% [49, 61, 63]. These studies suggested the potential of circulating antigens as useful biomarkers for the diagnosis of schistosomiasis mekongi.

## Diagnosis towards elimination

A key element to achieving elimination status of schistosomiasis is the diagnosis. Commonly used diagnostic tools, especially those utilized in control programs, are often lacking in sensitivity leading to underestimation of prevalence. This negatively affects the progress of the control program since the assessment is heavily reliant on the efficiency and accuracy of the diagnosis. Moreover, elimination can only be fully realized in the presence of highly sensitive and specific diagnostic tools. To verify elimination or transmission interruption of schistosomiasis, WHO requires a two-step/two-test strategy where a positive result by an initial highly sensitive diagnostic test (ideally with > 95% sensitivity and > 80% specificity) will be retested by a second highly specific test (ideally with > 93% sensitivity and > 98% specificity) to confirm the diagnosis [68].

The true prevalence of *S. mekongi* in its endemic regions is difficult to assess. This is in part due to the limited availability of prevalence data on human and animal transmissions from all its endemic foci. Currently, Cambodia and Lao PDR are still known to have cases of *S. mekongi* infection [17, 27]. As of 2018, Lao PDR recorded an average prevalence of 0.7%, with the sentinel sites having an overall prevalence of 3.2%. In Cambodia, data on sentinel sites showed a huge decrease in prevalence and remained below 1% in 2018 [27]. In Myanmar, a number of cases have also been reported in selected regions such as Bago and Rakhine, although precise mapping is needed to determine the actual status of the infection. On the other hand, Thailand is considered to be in transmission interruption pending verification by WHO [17, 70].

Most of the reported prevalence data from surveillance in these regions were based on Kato-Katz technique. Although considered the gold standard for diagnosing schistosomiasis, Kato-Katz lacks sensitivity and is not suitable for use in communities with low intensity of the infection [4, 7, 46, 48]. In communities from Cambodia and Lao PDR where high intensity of the infection is not being seen recently, false negatives are very likely to cause an underestimation of the actual prevalence [4, 60]. Furthermore, for schistosomiasis monitoring in regions nearing elimination or in regions under transmission and surveillance control programs, a more sensitive diagnostic approach is needed [5, 27]. This includes serologic antibody-based and molecular nucleic acid-based diagnosis [20, 35]. Serology-based technique promise high sensitivity and efficiency of use, but may yield false positive results and exhibit cross reactivity with other parasitic infections. Thanchomnang et al. used 18S ribosomal RNA gene-targeted conventional PCR coupled with pyrosequencing for identification and differentiation of *S. mekongi* from *S. japonicum* among cercariae from snails, and eggs from mice and rats’ samples. This protocol was highly sensitive, as it was able to detect the target DNA in spike samples of 10 healthy snails with one cercariae and 100 mg of feces with 2 eggs. It was also highly specific, as it did not amplify the DNA from other schistosome species [57].

In addition, RT-PCR and PCR-coupled techniques has been successfully applied in the surveillance of schistosome-derived DNA in the environment. Although not diagnostic in nature, the detection of which may indicate contamination and, therefore, prevent people from getting into contact with contaminated water and prevent them from getting infected. Using a fluorophore-labelled hybridization probes in real-time fluorescence resonance energy transfer (FRET) technology with subsequent melting-curve analysis, Sanpool et al. evaluated the use of RT-PCR in a detection system for *S. mekongi* in snail samples, fecal samples from infected rats, and water samples containing cercaria. Targeting the mitochondrial genome, the technique showed 100% sensitivity and specificity, and was able to confirm presence of a cercaria in one liter of water.

Nucleic acid-based diagnostics provide high accuracy, but it is costly and may require sophisticated instruments, controlled conditions, and personnel expertise. Interests and work in improving and standardizing these techniques for use in schistosomiasis diagnosis have increased in the past few years. One common strategy to improve accuracy and efficiency of diagnosis is to complement Kato-Katz with molecular or serologic tests as a way to improve sensitivity and specificity further [24].

With the apparent downward trend in the prevalence of *S. mekongi* infection in endemic countries, control programs are now focused on attaining transmission interruption and eventual elimination. To achieve this road map, the One Health approach, a multidisciplinary and multisectoral strategy developed by WHO, is currently being employed in Cambodia and Lao PDR [27, 51]. This strategy integrates community-led Water, Sanitation, and Hygiene (CL-WASH) interventions, MDA with PZQ, and surveillance with subsequent treatment of human hosts and animal reservoirs [17, 27, 69]. In this approach, community empowerment is emphasized and recognized as a vital factor in achieving sustainable control and development [69]. Testing of domestic animals at risk of *S. mekongi* infection is also given importance. Dogs and pigs are known reservoirs of *S. mekongi* and, thus, play a role in the continued transmission of the parasite [22].

## Conclusion

Compared to other schistosome species, elimination of *S. mekongi* infection could*,* in theory, be easier due to its limited distribution. Unfortunately, that is so much easier said than done. Still, with the continuous collaborative efforts of the local government units and communities supported by global research cooperation, elimination is a possibility. Central to these efforts is diagnosis. As the prevalence of *S. mekongi* infection goes down, the development of diagnostics must focus on improving sensitivity and accuracy to be able to detect all cases. An efficient diagnostic strategy will benefit control and surveillance programs resulting in proper assessment of the true prevalence of the disease, thereby leading to the eventual elimination of schistosomiasis.

## Data Availability

Not applicable.
